# Retinoid Differentiation Therapy for Common Types of Acute Myeloid Leukemia

**DOI:** 10.1155/2012/939021

**Published:** 2012-06-12

**Authors:** Geoffrey Brown, Philip Hughes

**Affiliations:** School of Immunity and Infection, College of Medical and Dental Sciences, University of Birmingham, Edgbaston, Birmingham B15 2TT, UK

## Abstract

Many cancers arise in a tissue stem cell, and cell differentiation is impaired resulting in an accumulation of immature cells. The introduction of all-*trans* retinoic acid (ATRA) in 1987 to treat acute promyelocytic leukemia (APL), a rare subtype of acute myeloid leukemia (AML), pioneered a new approach to obtain remission in malignancies by restoring the terminal maturation of leukemia cells resulting in these cells having a limited lifespan. Differentiation therapy also offers the prospect of a less aggressive treatment by virtue of attenuated growth of leukemia cells coupled to limited damage to normal cells. The success of ATRA in differentiation therapy of APL is well known. However, ATRA does not work in non-APL AML. Here we examine some of the molecular pathways towards new retinoid-based differentiation therapy of non-APL AML. Prospects include modulation of the epigenetic status of ATRA-insensitive AML cells, agents that influence intracellular signalling events that are provoked by ATRA, and the use of novel synthetic retinoids.

## 1. Introduction

There is an increasing need to devise milder treatments for older patients with cancer. The proportion of older people in the population is steadily increasing, and 23% of the UK population is projected to be aged ≥65 by 2034, with 5% aged ≥85 [1]. More than half of cancer patients are aged ≥65 years, and around 40% of older persons will be diagnosed with some form of cancer. Treatment of these patients poses a real challenge to the health care service, more so as the population as whole ages. For older persons, an immediate resort to eradicating cancer *via* aggressive chemotherapy is neither well tolerated nor necessary. Differentiation therapy to reduce tumour load followed by milder chemotherapy provides an alternative approach. It is also important to bear in mind that many patients aged ≥65 years are excluded from aggressive trials, by coexisting age-related conditions, for example, declining bone marrow [2, 3] and hepatic function [4]. Trials of new combinations of drugs in leukemia include only younger patients able to tolerate multidrug chemotherapy. For patients in their 80s with malignancies, differentiation therapy that might merely aim to control disease for the patient's natural lifespan is perhaps a more realistic target. 

AML, which accounts for ~80% of adult acute leukemias [5], involves the proliferation, abnormal survival, and arrest of cells at a very early stage of myeloid cell differentiation. Alongside this expansion of leukemia cells, the production of normal red cells, platelets, and neutrophils is reduced: these deficits are also life threatening as in the case of infections relating to neutropenia. Current cytotoxic chemotherapy for AML results in a remission rate of 60 to 80% for patients <60 years of age. However, most patients relapse with treatment-resistant disease, and 5-year survival rates are low around 30% [6, 7]. Survival is worse for patients >60 years of age, largely because these patients cannot tolerate intensive treatment and the ensuing further ablation of normal haematopoiesis [8–10]. Thirty-five percent of AML patients are aged ≥75 years, and the median age at diagnosis is 72 [11]. These patients are very hard to treat with current regimens, and whilst younger patients have benefited from more intensive approaches to treatment, there have not been substantial improvements to outcomes for the elderly [12]. Only around 5% of elderly patients survive long-term when treated by conventional means [13], and stem cell transplantation is not an option for many patients [14]. 

There is a pressing need to improve survival rates between 5 to 30% and particularly the outcome for elderly patients which has not changed much during the last 20 years [8]. One of the reasons why current chemotherapies for AML are failing is that in endeavoring to eradicate the leukemia cells normal haematopoiesis is compromised substantially, with patients requiring blood and platelet transfusion. Hence, there is a compelling case for persevering with the development of new therapies that target the failure of AML cells to differentiate, are more efficacious in this regard, and have low haematological toxicity. Though AML is somewhat a rare malignancy, the various subtypes of AML provide an excellent test bed for new differentiation therapies, and proven new regimens might have a beneficial effect in treating more prevalent cancers that are presently incurable. 

## 2. The Success of ATRA in Acute Promyelocytic Leukaemia

Retinoids are a class of naturally occurring compounds that are structurally related to vitamin A (or retinol). Retinoids regulate a wide range of biological processes, including development, differentiation, proliferation, and apoptosis [15]. ATRA is the active metabolite of vitamin A and mediates its biological effects by activating one or more of the closely related retinoic acid receptors (RAR*α*, RAR*β*, and RAR*γ*) that function as ligand-dependent transcriptional regulators. These receptors form heterodimers with rexinoid receptors (RXR*α*, *β*, and *γ*) and bind to retinoid responsive response elements (RAREs) located in the promoter region of retinoid target genes to stimulate gene transcription [15]. Primitive human haematopoietic cells, such as CD133^+ve^/CD34^+ve^/lineage^−ve^ cells that are used to restore haematopoiesis after ablation of leukemia cells, express RAR *α*1, *α*2, and *γ*1. It is well known that activation of RAR*α* drives the differentiation of normal myeloid progenitor cells and myeloid cell lines towards neutrophils, and examination of *in vitro* models of ATRA-driven myeloid differentiation has identified genes that play important roles in this process, including transcription factors and regulators of survival versus apoptosis [16]. 

APL accounts for around 5–10% of cases of AML. This subtype of AML is characterised by cells having a promyelocytic morphology [17] and the chromosome translocation t(15; 17), resulting in fusion of the retinoic acid receptor *α* (RAR*α*, on chromosome 17) and promyelocytic leukemia (PML, on chromosome 15) genes [18]. The RAR*α* gene fuses with other genes in variants of APL, for example, the promyelocytic leukemia zinc finger gene (PLZF, on chromosome 11) as a result of t(11; 17) to generate PLZF-RAR*α* [19]. The RAR*α* fusion proteins block differentiation at the promyelocytic stage, by disrupting wild-type RAR*α* cellular signalling and promoting survival of myeloid precursor cells [20]. Cells from APL patients are exquisitely responsive to induction of cell differentiation by ATRA by virtue of this treatment leading to degradation of the RAR*α*/PML. ATRA combined with chemotherapy is a longstanding and highly successful way of treating APL. This has been well documented [21–25] and will only be described briefly here.

Treatment of APL patients with ATRA- and anthracycline-based chemotherapy results in cure rates up to 80%. Patients who relapse (up to 15%) receive intensive chemotherapy, with or without ATR, and around 90% of patients achieve a second remission. However, in most cases this is not durable and allogeneic or autologous stem cell transplantation is then recommended [26–28]. Arsenic trioxide drives rapid degradation of RAR*α*/PML within APL cells and induces differentiation and apoptosis and inhibits the proliferation of a variety of neoplastic cells [29–31]. In 2002, arsenic trioxide was introduced to treat APL patients who had relapsed and refractory APL, and more recent studies have revealed the effectiveness of arsenic trioxide as a primary and single curative agent [32–34].

ATRA has been highly successful in the treatment of APL, but the promise of extending the efficacy of ATRA-based differentiation therapy to other types of AML, and other leukemias and cancers, has still to be fulfilled. In the following sections we examine some of the recent research strategies that are seeking to extend and improve differentiation therapy for AML.

## 3. Aberrant Epigenetic Gene Regulation and Unresponsiveness of AML Cells to ATRA

One of the reasons why non-APL AML cells respond poorly to ATRA is that target genes that are important to the ATRA-driven differentiation pathway are not properly activated as to their transcription. In the case of non-APL AML cells, ATRA fails to activate the *RARA2* gene which is important for differentiation [35]. Also, RAR*α*2 expression is reduced in AML cells, relative to normal CD33^+ve^ cells, and this is related to a diminution in histone H3K4 dimethylation (H3K4^me2^) on the *RARA2* gene promoter [36], whereby dimethylation is associated with activation of transcription. The H3K4me1/me2 lysine-specific demethylase 1 (LSD1/KDMI) is highly expressed in AML cells [37] and various other tumour cells [38, 39]. ASXL1, a cofactor for RAR, recruits LSD1 to repress RAR target gene promoters. 

Recently, Zelent and coworkers have examined the importance of LSD1 in the lack of responsiveness of non-APL AML cells to ATRA [40]. Inhibitors of LSD1 include tranylcypromine (*trans*-2-phenylcycloprolamine) [41] and the biguanide polyamine analogue 2d (1,15-bis{N5-[3,3-(diphenyl)propyl]-N1-biguanido}-4,12-diazapentadecane) [42]. Tranylcypromine is an antidepressant (sold under the brand names Parnate and Jatrosom) and is well tolerated. Inhibitors of LSD1 and ATRA synergised to drive differentiation of primary human AML cells and enhance H3 lysine-4 dimethylation and the expression of myeloid differentiation-associated genes. ATRA and inhibitors of LSD1 when used alone had a limited effect on primary AML cells. Knockdown of LSD1 (shRNA) in HL-60 and TEX (human cord blood immortalised by expression of the *TLS-ERG* oncogene) cells confirmed that this enzyme attenuates the responsiveness of AML cells to ATRA. Importantly, ATRA plus tranylcypromide target leukemia-initiating cells as revealed by diminished engraftment when primary AML samples were treated with the two agents before and after transplantation into NOD.*SCID* gamma mice. The ability of inhibitors of LSD1 to restore responsiveness of non-APL AML cells to ATRA and the anti-leukemic effect of ATRA in combination with an inhibitor of LSD1 indicate a promising new way forward to differentiation therapy of non-APL AML.

DNA methylation at certain gene promoter regions, by DNA methyltransferase enzymes adding methyl groups to CpG sites, may contribute to leukemogenesis by silencing tumour suppressor genes [43, 44]. Such aberrant gene silencing may also be more common in older persons [45]. The use of inhibitors of DNA methyltransferases in the treatment of myelodysplastic syndromes and AML has focused on the nucleoside analog of cytidine azacitidine. The US Food and Drug Administration has approved the use of this agent to treat myelodysplastic syndromes and treatment of patients with higher-risk myelodysplastic syndrome with azacitidine results in a significant survival advantage as compared with conventional care regimens [46]. Azacitidine also prolongs overall survival in elderly AML patients with a low blast count in their bone marrow [47], and treatment of elderly AML patients with outpatient azacitidine resulted in a response rate of around 20% [48]. 

Lenalidomide, an immunomodulatory agent approved for use in myelodysplastic syndromes and myeloma [49], has epigenetic modifying properties [50] and appears to upregulate tumour suppressor genes that are activated by azacitidine [51]. Treatment of high-risk myelodysplastic syndrome patients with a combination of azacitidine and lenalidomide resulted in a complete response in 44% of patients [52]. Recently, Pollyea and co-workers have examined the prospect of using azacitidine and lenalidomide sequentially to induce remission in elderly and previously untreated patients with AML [53]. Eighteen patients received treatment to determine safety, efficacy, and biological predictors of response. Marked genome-wide DNA demethylation occurred, and ten of the sixteen evaluable patients responded with seven patients achieving a complete remission or remission with incomplete recovery of blood counts. These results as to biological and clinical activity are very promising, and the extent to which sequential azacitidine and lenalidomide will be beneficial in elderly and untreated AML patients, and such patients with a low disease burden, awaits the outcome from an ongoing phase 2 study. 

Epigenetic therapies are an important consideration as to rationales for the induction and maintenance of responses in elderly AML patients and for treatments that are tolerable. There is still more to unravel in the use of histone demethylase inhibitors, to drive expression of myeloid differentiation-associated genes, and/or inhibitors of DNA methyltransferases, to drive growth arrest. Interestingly, lenalidomide-provoked epigenetic modifications appear to involve a LSD1-mediated process [49]. Lenalidomide provokes cell cycle arrest in cell lines that typify Burkitt's lymphoma and multiple myeloma by increasing the level of expression of p21(WAF-1), and transcription factors that bind to CpG-rich promoter regions are involved in this process. Lenalidomide-induced up regulation of p21(WAF-1) was reduced by silencing of LSD1, suggesting the involvement of this lysine-specific histone demethylase in a priming switch from methylated to acetylated H3 on the p21(WAF-1) promoter. 

## 4. Improving ATRA Sensitivity by Inhibiting the Activity of an Aldoketoreductase

ATRA can be used to drive differentiation of non-APL myeloid leukemia cell lines, but this often requires a much higher concentration to obtain the same degree of differentiation as APL cell lines. One way in which the concentration of ATRA required for differentiation of both APL and non-APL cells can be reduced is by inhibiting the activity of the aldoketoreductase AKR1C3. Our laboratory first became interested in this enzyme following observations that inhibition of AKR1C3 with the nonsteroidal anti-inflammatory drug indomethacin or the progestogen medroxyprogesterone acetate (MPA) increased the responsiveness of the human promyeloid cell line HL-60 to both ATRA and 1*α*,25-dihydroxyvitamin D_3_ [54, 55] and that AML cell lines express AKR1C3 at a high level [56]. Overexpression of AKR1C3 in HL-60 cells led to resistance to ATRA- and 1*α*,25-dihydroxyvitamin D_3_-mediated differentiation, confirming the enzyme as a novel regulator of nuclear receptor-regulated cell differentiation [57]. 

AKR1C3 is a multifunctional NADPH-dependent oxidoreductase that plays a role in the metabolism of androgens, oestrogens, prostaglandins, retinoids, and xenobiotics [58]. Hence, AKR1C3 can potentially control the supply of ligands to several classes of nuclear hormone receptors that modulate the survival, proliferation, and differentiation of hematopoietic cells. There is also evidence to implicate AKR1C3 in leukemogenesis. Activating polymorphisms of the AKR1C3 gene has been associated with an increased chance of developing childhood myeloid leukaemia [59], and increased expression of AKR1C3 has been observed in a patient with myelodysplastic syndrome who went on to develop AML-M2 [60]. Workers in the petrochemical industry have a higher-than-normal risk of developing myeloid leukemia, and smoking is a risk factor for myelodysplatic syndromes and AML [61]. A main factor appears to be an increased production of carcinogenic activated polycyclic aromatic hydrocarbon metabolites which cause oxidative DNA damage and DNA strand breakage. Birtwistle and co-workers [62] have recently shown for primary AML cells and in a model system that elevated AKR1C3 expression leads to conversion of model polycyclic aromatic hydrocarbons into compounds that induce DNA damage.

**Figure 1:**
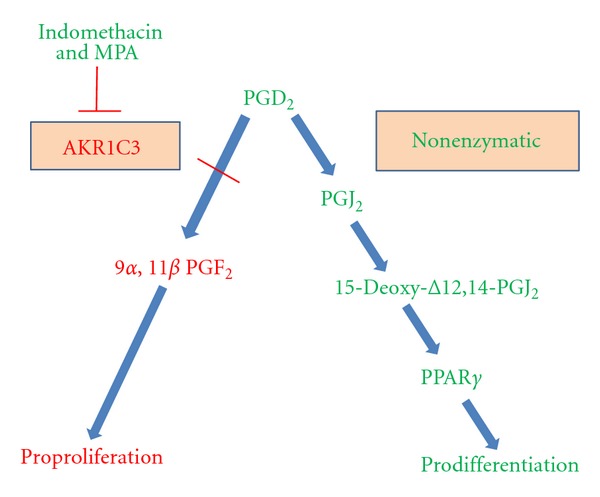
Influence of inhibiting the activity of AKR1C3 on PGD_2_ metabolism. Inhibiting the activity of AKR1C3 by the use of indomethacin or medroxyprogesterone acetate (MPA) interferes with prostaglandin D_2_ (PGD_2_) metabolism towards 9*α*,11*β* PGF_2_ and favours nonenzymatic metabolism towards J-series prostanoids and the PPAR*γ* ligand 15-deoxy-Δ12,14-PGJ_2_.

AKR1C3-mediated metabolism of prostaglandins (PGDs) provides a rationale to the influence of this enzyme on cell differentiation (Figure 1). PGD_2_ is a substrate for AKR1C3, due to its 11-ketoprostaglandin reductase activity, and would be preferentially metabolised to its 9*α*,11*β*-epimer PGF_2_ [58], which enhances proliferation of several myeloid leukemic cell lines. However, endogenous PGD_2_ is also relatively unstable and will be rapidly and efficiently non-enzymatically converted first to PGJ_2_ and then, in a stepwise manner, to 15-Deoxy-Δ12,14-PGJ_2_. 15-deoxy-Δ12,14-PGJ_2_ is a ligand for the peroxisome proliferator-activated receptor-*γ* (PPAR*γ*) and can suppress cell proliferation and enhance differentiation of myeloid leukemic cells [57]. Desmond and co-workers [57] have shown for myeloid progenitor cells that express a high level of AKR1C3 that PGD_2_ catabolism can be switched from the generation J-series prostanoids that would enhance differentiation and suppress proliferation towards the production of the pro-proliferative PGF_2_.

The PPAR*γ* ligand troglitazone can sensitize HL60 cells to the differentiating and anti-proliferative effects of ATRA and 1*α*,25-dihydroxyvitamin D_3_ [63]. However, there are concerns about toxicity associated with high-dose PPAR*γ* agonist therapy. Fibrates such as clofibrate and bezafibrate are agonists of PPAR*α* [64, 65] and modest potentiating agents. Importantly, fibrates have a good toxicity profile and are well tolerated by patients. In this regard, Murray and co-workers have used a combination of bezafibrate (to agonise PPAR*α*) and medroxyprogesterone acetate (to inhibit AKR1C3) to treat a small number of elderly patients with myelodysplastic syndrome and AML [66]. Improvements in the hematological profile were observed, and there were limited signs of toxicity. Whether the addition of ATRA would further improve the therapeutic outcome has still to be examined.

AKR1C3 may have a direct affect on ATRA-provoked cell differentiation by lowering the intracellular concentration of ATRA. Low cellular levels of ATRA have recently been shown to be a feature of a variety of malignant cells [67]. In HL-60 cells AKR1C3 can act as a retinaldehyde reductase, to promote conversion of retinaldehyde into retinol and, as such, decrease the level of cellular ATRA [68]. Ruiz and co-workers have suggested that activity of AKR1C3 plays a role in driving proliferation of HL-60 cells: this can be blocked by a combination of an AKR1C3 inhibitor and a retinoid acid receptor antagonist [68]. The pro-proliferative retinoid signal might well be an extremely low concentration of endogenous ATRA, provoked by activity of AKR1C3, and for the following reasons. When HL-60 and NB4 cells are grown serum-free (that is free of exogenous retinoid), sub-nanomolar concentrations of ATRA enhance colony formation by single cells and the proliferation of cells in bulk cultures [69–71]. At very low ATRA concentrations it is likely that any RAR signalling is mediated *via* preferential activation of RAR*γ*, favouring cell proliferation/survival as opposed to differentiation [72, and see later novel synthetic retinoids]. In this case and as observed by Ruiz and co-workers, an antagonist of RAR, particularly of RAR*γ*, would be expected to interfere with cell growth [73]. As to this possible mode of action of AKR1C3, again inhibiting enzyme activity is important for increasing the sensitivity of myeloid leukemia cells to ATRA.

## 5. Potentiating ATRA-Stimulated ****Differentiation by Inhibiting Glycogen ****Synthase Kinase

As the name suggests, the constitutively active serine/threonine kinase glycogen synthase kinase 3 (GSK-3) plays a role in glycogen biosynthesis and insulin action by phosphorylating and inactivating glycogen synthase. However, GSK-3 is now known to phosphorylate a wide range of proteins and play a role in intracellular signalling that is initiated by various stimuli, as GSK-3 is functionally inactivated when phosphorylated *via* the growth factor receptor-activated RAS-MAP kinase, ERK5/RSK-2 and PI3K-PKB/AKT signalling pathways [74]. Therefore, compounds that inhibit the activity of GSK-3 are very likely to affect many biological processes. Of importance to differentiation therapy is that inhibitors of GSK-3 appear to have opposing effects on the proliferation and be survival and commitment to differentiation of normal and leukemic hematopoietic stem cells (HSCs). This may provide a treatment that targets transformed cells and spares their normal counterpart.

Pharmacological inhibition or genetic depletion of GSK-3 has been associated with increased self-renewal and reduced commitment to differentiation of HSCs in normal mice, and Huang and co-workers have shown that GSK-3 activity inhibits signalling through the WNT pathway to enhance lineage commitment of HSC [75]. In transformed hematopoietic cells, the situation appears to be different. Overexpression and overactivation of GSK-3 are associated with an unfavorable prognosis in AML [76]. Sustained proliferation of MLL-transformed leukemia cells is dependent on activity of GSK-3, leading Wang and co-workers [77] to propose that GSK-3 acts as a tumor promoter in this model system. In keeping, treatment of MLL-transformed cells *ex vivo* with inhibitors of GSK-3 led to arrest of growth in G1 and an increase in the rate of spontaneous differentiation towards myeloid cells. GSK-3 inhibitors also enhanced the survival of mice with these leukemias. In a follow-up study, Wang and co-workers showed that GSK-3 controls the formation of a HOX/MEIS1/CREB complex which recruits the coactivators CBP and TORC to form a molecular complex that appears to promote self-renewal and survival of the transformed cells [78]. The transformed cells were driven into apoptosis following treatment with the GSK-3 inhibitor lithium chloride. Other workers have confirmed that leukemic HSCs are more sensitive to induction of apoptosis by GSK-3 inhibitors than normal HSCs [79, 80].

The possible use of GSK-3 inhibitors in differentiation therapy for acute myeloid leukemia dates back to around 1993. Sartorelli's group showed that high concentrations of lithium chloride induced growth arrest and myeloid differentiation of the murine myelomonocytic cell line WEHI-3B D+ and HL-60 cells [81]. In the case of HL-60 cells, lithium chloride induction of neutrophil differentiation was markedly enhanced by the addition of a low amount of ATRA, and no such interaction was seen with agents that drive HL-60 cells to differentiate towards monocytes [81]. These findings have been revisited in recent years, and several structurally unrelated GSK-3 inhibitors have now been shown to potently inhibit the growth and drive differentiation of a variety of primary leukemic cells and leukemic cell lines [82–87]. Importantly, genes that are upregulated (Id1, *C⁄*EBP*ε*, Stat1, p21, and p27) or downregulated (CDK8, c-myc) during ATRA-stimulated myeloid differentiation of HL60 and NB4 cells and whose products are important for cell differentiation, are similarly regulated by inhibitors of GSK-3 [86, 87]. 

**Figure 2:**
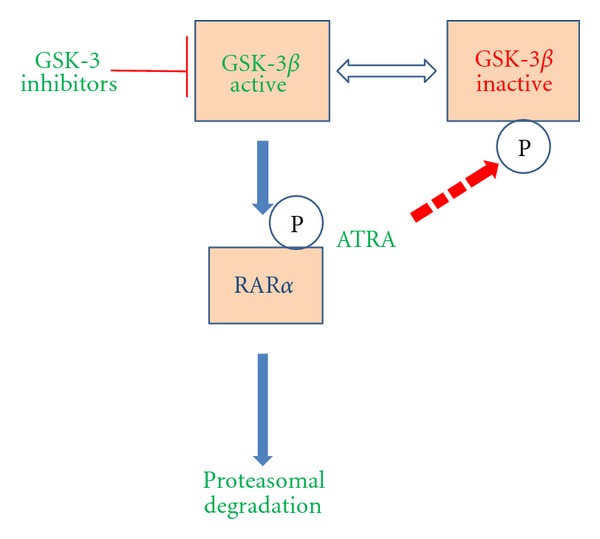
Inhibition of the activity of GSK-3*β* is important for ATRA sensitivity, The figure shows one way in which activity of GSK-3*β* affects ATRA responsiveness of myeloid cells and how transcriptional activity of RAR*α* and kinase activity of GSK-3*β* are linked *via* ATRA-related inhibitory phosphorylation (P) of GSK-3*β*.

Levels of RAR*α* are linked to the activity of GSK-3*β*. Sartorelli's group has shown that inhibitors of GSK-3 prevent ATRA-mediated degradation of RAR*α*, thereby potentiating ATRA-stimulated differentiation of AML cell lines [88, 89]. A recent study provides a molecular basis for this observation. Si and co-workers have shown that GSK-3*β* phosphorylates RAR*α* on multiple serine residues, and phosphorylation of RAR*α* reduces its transcriptional activity. GSK3 phosphorylation of certain proteins enhances their proteasomal degradation, and it is possible that this is the case for RAR*α* as treatment of myeloid leukemia cells with GSK-3 inhibitors led to enhanced expression of RAR*α* [86]. Accordingly, GSK-3 inhibitors potentiated RAR*α*-mediated transcriptional activity which could be explained by increased expression of RAR*α* and by GSK-3*β* inhibitor-mediated upregulation of the expression of the RAR transcriptional coactivators p300, SRC-1, and CBP [86]. Transcriptional activity of RAR*α* and kinase activity of GSK-3*β* are also linked as follows (Figure 2). In the retinoid-sensitive AML lines HL-60 and NB4, but not the retinoid insensitive K562 cell line, ATRA treatment was associated with a time- and concentration-related phosphorylation of an N-terminal serine of GSK-3*β* [86]. This inhibitory phosphorylation of GSK-3*β* is an early step in the ATRA-induced differentiation of myeloid leukemia cells, in keeping with the importance of inhibition of activity of GSK-3*β* to cell differentiation.

The GSK-3 inhibitors lithium chloride and valproate are already in clinical use, and the above studies indicate that a combination of these agents and a RAR*α* agonist may enhance the effectiveness of differentiation therapy for both ATRA-sensitive and insensitive AML. In addition, beneficial effects from inhibiting the activity of GSK-3*β* might also relate to the involvement of this enzyme in sustaining proliferation of leukemia cells (see above). 

## 6. Potentiation of ATRA-Stimulated ****Differentiation of Myeloid Leukemic Cells ****by Inhibitors of Inosine 5′-Monophosphate Dehydrogenase

Inosine 5′-monophosphate dehydrogenase (IMPDH) is the rate limiting step in the *de novo* synthesis of guanine nucleotides. This enzyme catalyses NAD^+^-dependent oxidation of inosine 5′-monophosphate to xanthosine 5′-monophosphate, which is subsequently aminated to guanosine-5′-monophosphate which is converted to guanosine triphosphate (GTP) and deoxyguanosine triphosphate (dGTP). As such, IMPDH plays an important role in the maintenance of the intracellular levels of guanosine nucleotides; especially GTP, and GTP, and dGTP are essential for the synthesis of DNA and RNA. It has long been known that depletion of guanosine nucleotides accompanies differentiation of a variety of cell types. For example, myeloid differentiation of HL-60 and other myeloid leukemic cells is accompanied by a reduction in the cellular GTP content and differentiation can be blocked by the addition of exogenous guanosine [90]. IMPDH activity and GTP levels appear to be much higher in leukemic blast cells than in their normal counterparts, which might contribute to the failure of blast cells to complete their differentiation programme. 

Twenty-five years ago Knight and co-workers [91] showed that ATRA-mediated myeloid differentiation of HL-60 and RDFD2-25 myeloid leukemic cells was associated with a rapid decrease in IMPDH activity. A fall in IMPDH activity was not observed in a retinoid-insensitive variant of the RDFD2-25 cell line, which suggests that the fall in IMPDH activity is an important aspect of the differentiation process. Commensurate with this finding is that depletion of intracellular GTP levels *via* the use of a variety of IMPDH inhibitors, such as mycophenolate mofetil, tiazofurin and 3-hydrogenkwadaphnin, leads to differentiation and/or apoptosis of myeloid leukemic cell lines [92–94]. Also, treatment of HL-60 and NB4 cells with a combination of ATRA and an IMPDH inhibitor resulted in a greater degree of neutrophil differentiation, followed by apoptosis, than when either agent was used alone [95, 96]. Overall, these studies suggest that a combination of IMPDH inhibitors (e.g., mycophenolate mofetil), which are already in clinical use, with ATRA may provide a more effective treatment for both APL and non-APL AML.

## 7. Enhancing ATRA Responsiveness by ****Inhibiting Telomerase Activity

Recent studies suggest that sensitivity of cell lines that typify APL to the combination of ATRA and arsenic trioxide is due to a synergy at the level of inhibition of telomerase activity. Myeloid leukemic stem and progenitor cells exhibit a higher degree of telomerase activity than their normal counterparts which may contribute to their increased capacity for self-renewal and reduced sensitivity to differentiating agents. Shortened telomere length and elevated telomerase activity in cells from APL patients are indicative of extensive proliferative capacity and correlate with disease progression and relapse. Thus, elevated telomerase activity may serve as prognostic factors for a subset of APL patients with more aggressive disease and poor outcome and those who may not respond favourably to arsenic therapy [97].

Overexpression of telomerase in normal HCSs changes these cells into ones resembling a leukemic stem cell, suggesting a role for telomerase in the leukemogenic process. The catalytic subunit of telomerase is telomerase reverse transcriptase (TERT). In the case of HL-60 cells, a decrease in the expression of the hTERT gene and a concomitant reduction in telomerase activity is a relatively early event following exposure to differentiating concentrations of ATRA. Genetic knockdown of hTERT is sufficient to induce growth arrest and eventually drives HL-60 cells into apoptosis [98–102]. Downregulation of hTERT expression following retinoid treatment has also been observed in maturation-resistant APL cell lines and non-APL AML cell lines, but in these cell lines cotreatment with a specific RAR*α* agonist and a retinoid X receptor- (RXR-) specific agonist (a rexinoid) was required. In non-APL AML cells there was no indication of an increased level of granulocytic differentiation following retinoid/rexinoid treatment, suggesting that retinoid/rexinoid-mediated down-regulation of telomerase was targeting a pathway that is important for survival [103, 104]. That down regulation of hTERT gene expression appears to be a key early event in ATRA-mediated growth arrest, and apoptosis induction of both retinoid-sensitive and insensitive APL cell lines and non-APL myeloid leukemic cells, suggests that a combination of ATRA (± a rexinoid) with telomerase inhibitors may have enhanced antileukemic properties in APL patients and also be beneficial in non-APL AML.

## 8. The Use of Novel Synthetic Retinoids

A complication during ATRA treatment of APL is retinoic acid syndrome (RAS). The full-blown syndrome is life-threatening as patients may develop renal failure or respiratory distress and require admission to intensive care. At the earliest sign of RAS, treatment with intravenous dexamethasone is the recommended course of action, and ATRA is temporarily discontinued in the case of severe RAS [21, 105]. Respiratory distress and fever can be very common; in a retrospective analysis of 102 APL patients who received ATRA as an induction regimen with or without conventional chemotherapy, 87.5% of patients developed respiratory distress and fever [106].

ATRA is promiscuous as regards binding to retinoid receptors. ATRA binds with high affinity to the *α*, *β*, and *γ* sub-types of RARs, and to RXRs by virtue of isomerising within cells to 9-*cis*-retinoic acid. As to the importance of ligand-activation of RAR*α* for myeloid cell differentiation [107], synthetic retinoids have been developed that only bind to this RAR sub-type. Even though there are toxicities associated with ATRA, the success in treating APL somewhat precludes using a RAR*α*-specific agonist instead of ATRA. However, a RAR*α* specific agonist and also synthetic antagonists of RARs are worthy of consideration for future new therapies.

We and others have examined the biological activity of isoform-selective synthetic agonists and antagonists of RARs with a view to widening the scope as to the use of retinoids to drive growth arrest, differentiation, and/or apoptosis of malignant cells [108, 109]. The extents to which the retinoids we have studied over a number of years are selective for a particular receptor subtype(s) are shown in Table 1.

As shown in Table 1, the compound AGN195183 is a highly selective agonist of RAR*α*; it binds to RAR*α* with a low nanomolar affinity (ED_50_ 20.1 nM), affinities to *RARβ* and *γ* are much higher (ED_50_ > 5,000), and it does not bind to RXRs (ED_50_ > 10,000). Treatment of HL-60 cells with the RAR*α* agonist, rather than agonising all RARs, is sufficient to drive differentiation towards neutrophils [108]. Hence, AGN195183 is suitable for use in differentiation therapy of AML and might circumvent some of the toxicities that are associated with the use of ATRA.

Antagonising, rather than agonising, RARs may be important for the development of milder treatments for elderly patients with leukemia and other cancers. Some time ago we showed that antagonising all RARs (AGN194310) inhibited the growth of patients' prostate cancer cells more effectively than normal prostate epithelium. Treatment of prostate cancer cells in liquid culture led to growth arrest in the G1 phase of the cell cycle followed by apoptosis. Moreover, the pan antagonist appears to be effective against tumor-initiating cells as nM concentrations of compound inhibited colony formation on plates by the small fraction of clonogenic cells [73, 110, 111]. Recently, we have shown that antagonising RAR*γ* is sufficient to inhibit the growth of prostate cancer cells [*manuscript submitted*]. In keeping with this perceived importance of RAR*γ* for survivability and/or proliferation of primitive cells, Purdon and coworker have shown that activation of RAR*γ* plays a role in self-renewal of HSC as these cells are reduced in RAR*γ*-null mice [72]. The use of a demethylase inhibitor (that is in clinical use and which restores ATRA responsiveness), plus an RAR*α* agonist (for differentiation) and an RAR*γ* antagonist (to inhibit growth) or the inhibitor plus either single retinoid, is interesting considerations to enhance the efficacy of differentiation therapy for ATRA-unresponsive AML.

## 9. Concluding Remarks

Healthcare authorities are already considering how to provide an appropriate standard of care for a population that in the future will overall be much older. A key aspect to this issue is the treatment of leukemia and other malignancies, particularly the extent to which elderly patients are able to tolerate current intensive therapeutic regimens. Differentiation therapy, and combined with less-aggressive chemotherapy, may provide milder treatments, and as outlined above there are promising ways forward for rendering differentiation therapy more efficacious. 

## Figures and Tables

**Table 1:** Preferential binding of synthetic retinoid analogs to subtypes of RAR. The equilibrium binding affinities of each retinoid analog were determined against baculovirus expressed RAR or RXR isoforms by displacement of [^3^H]-ATRA.

Binding affinities (ED_50_ in nM) against RARs and RXRs				
Retinoid analog	RAR*α*	RAR*β*	RAR*γ*	RXRs
RAR agonists				
Pan-RAR (AGN191183)	15.7	7.2	6.7	9,113 (*α*)–2,556 (*γ*)
RAR*α* (AGN195183)	20.1	>5,000	>5,000	>10,000
RAR*βγ* (AGN190168)	>1,000	14.2	135	>10,000
RAR*γ* (AGN205327)	3,766	734	32	>10,000
RAR antagonists				
Pan-RAR (AGN194310)	4.3	5	2	>10,000
RAR*α* (AGN196996)	3.9	4,036	>10,000	>10,000
RAR*βγ* (AGN194431)	300	6	20	>10,000
RAR*γ* (AGN205728)	2,400	4,248	3	>10,000
